# Supplementary material for: Proteomic characterisation of the Chlamydia abortus outer membrane complex (COMC) using combined rapid monolithic column liquid chromatography and fast MS/MS scanning
Source: PLoS One. 2019 Oct 24;14(10):e0224070. doi: 10.1371/journal.pone.0224070 (PMC6812762; doi:10.1371/journal.pone.0224070)
Supplement: S1 Fig — Predicted signal peptides, passenger domains, middle domains and autotransporter domains are highlighted in red, yellow, blue and green, respectively. All unique confidently identified peptides are in red, vertical lines indicate separation of adjacent peptides. Potential cleavage is indicated by gaps and a scissor symbol between domains in the protein figure below each amino acid sequence. Where there are no gaps between domains plus a scissor symbol this indicates the potential for cleavage but this was not supported by the data presented in this study. (PPTX) [file pone.0224070.s001.pptx]

## Slide 1
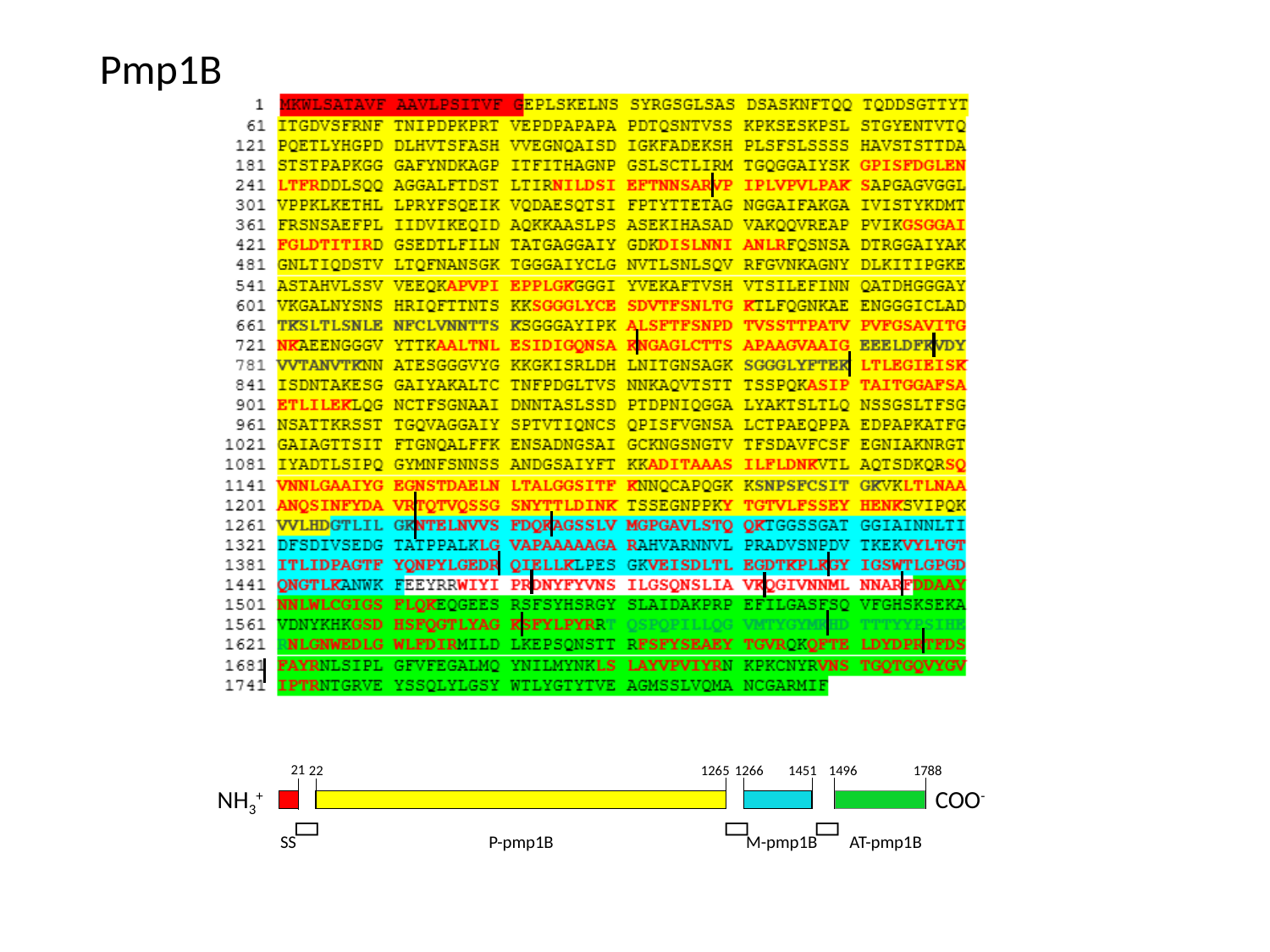

Pmp1B
21
1788
1496
1266
1451
1265
22
NH3+
COO-



SS P-pmp1B M-pmp1B AT-pmp1B

## Slide 2
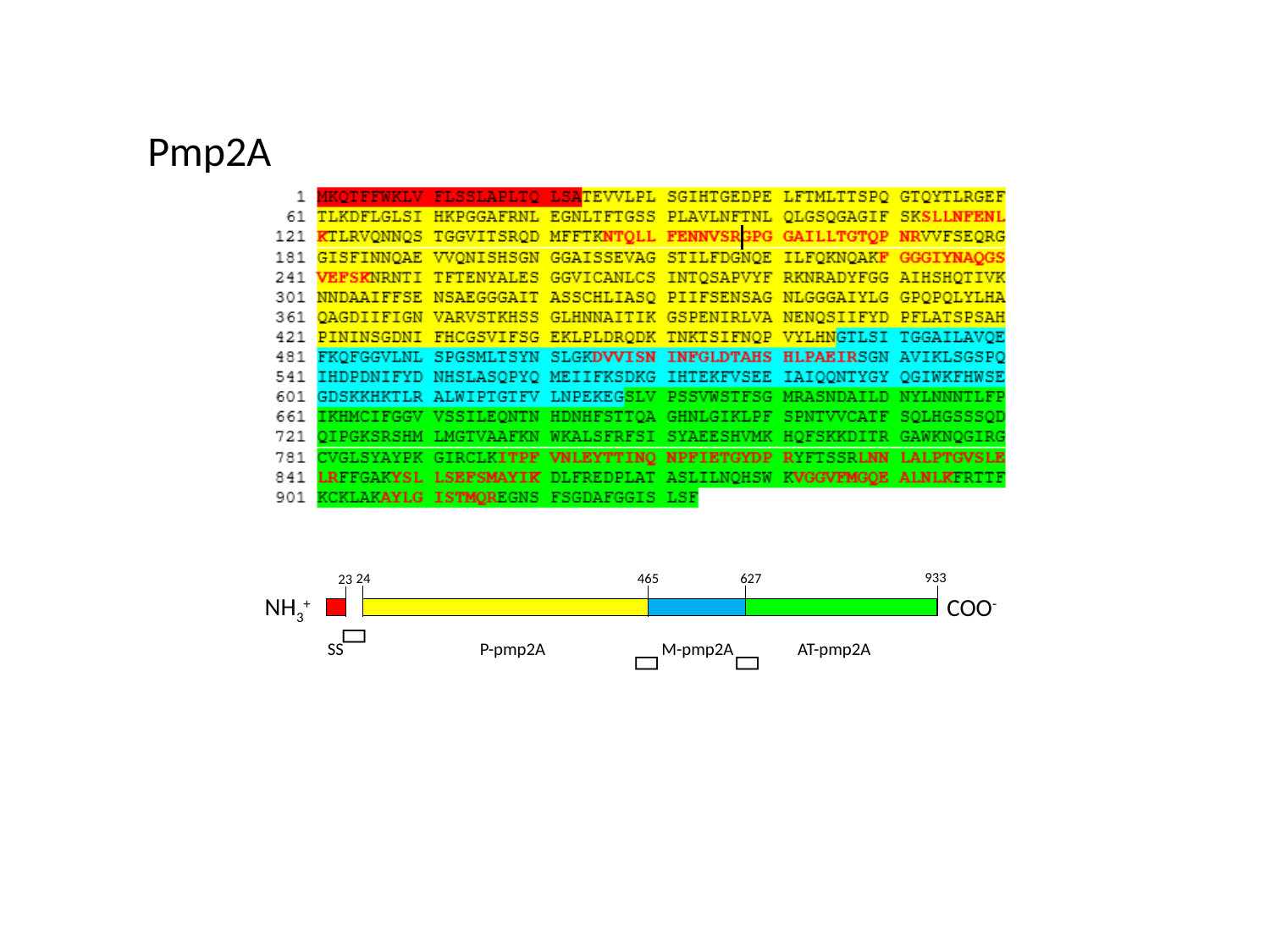

Pmp2A
933
627
465
24
23
NH3+
COO-



SS P-pmp2A M-pmp2A AT-pmp2A

## Slide 3
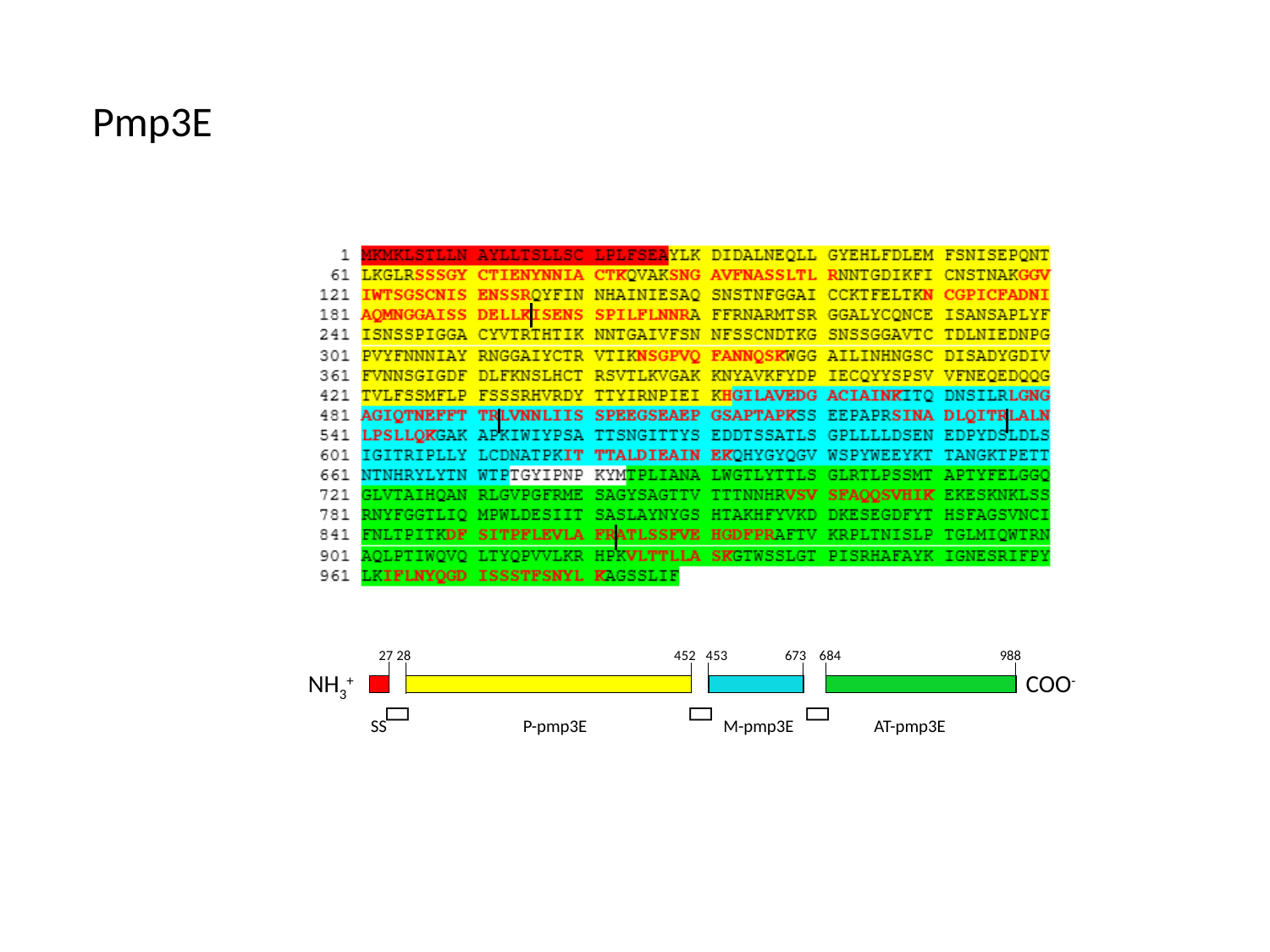

Pmp3E
988
684
453
673
452
27
28
NH3+
COO-



SS P-pmp3E M-pmp3E AT-pmp3E

## Slide 4
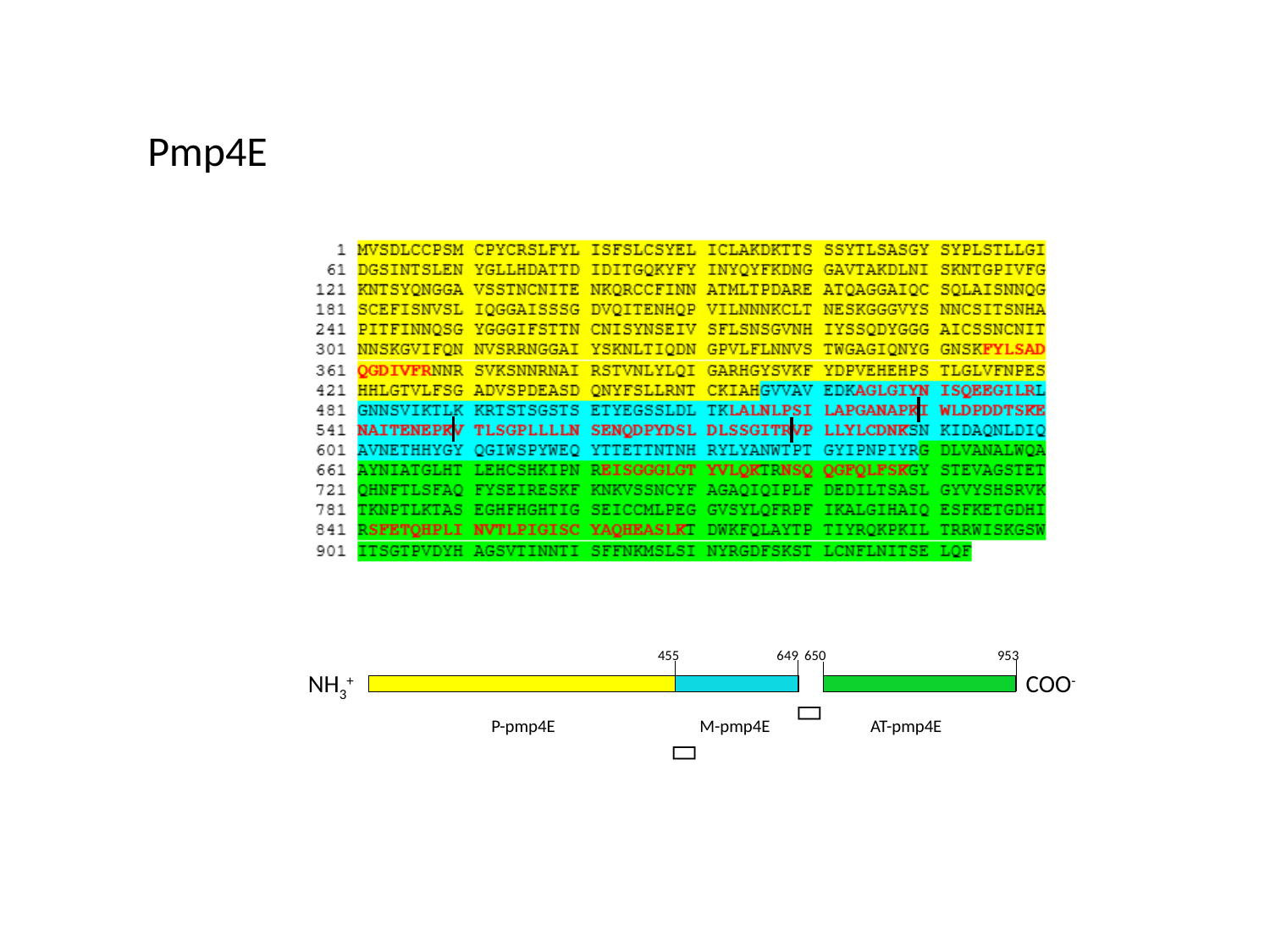

Pmp4E
953
649
455
NH3+
COO-

 P-pmp4E M-pmp4E AT-pmp4E

650

## Slide 5
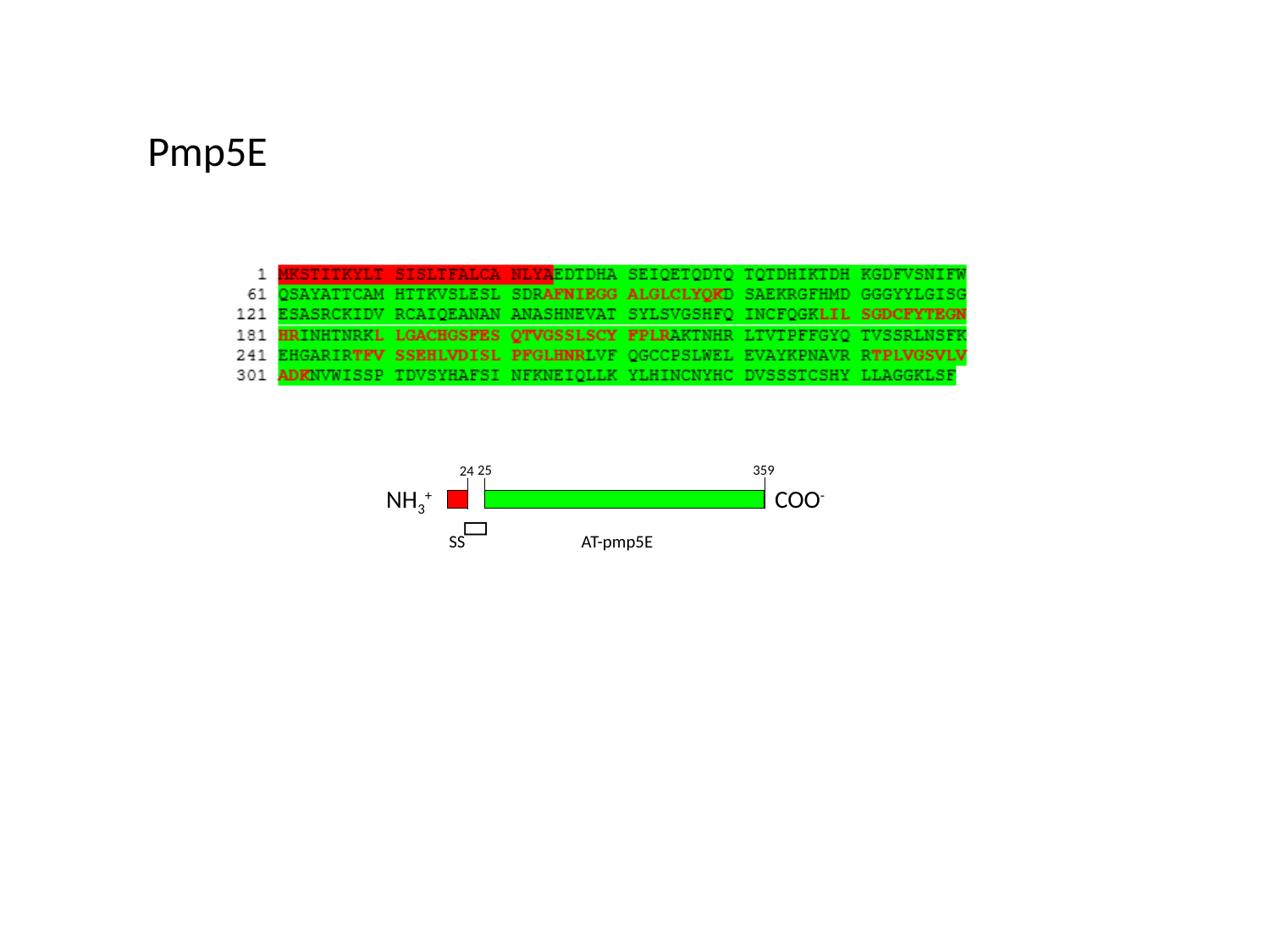

Pmp5E
359
25
24
NH3+
COO-

SS AT-pmp5E

## Slide 6
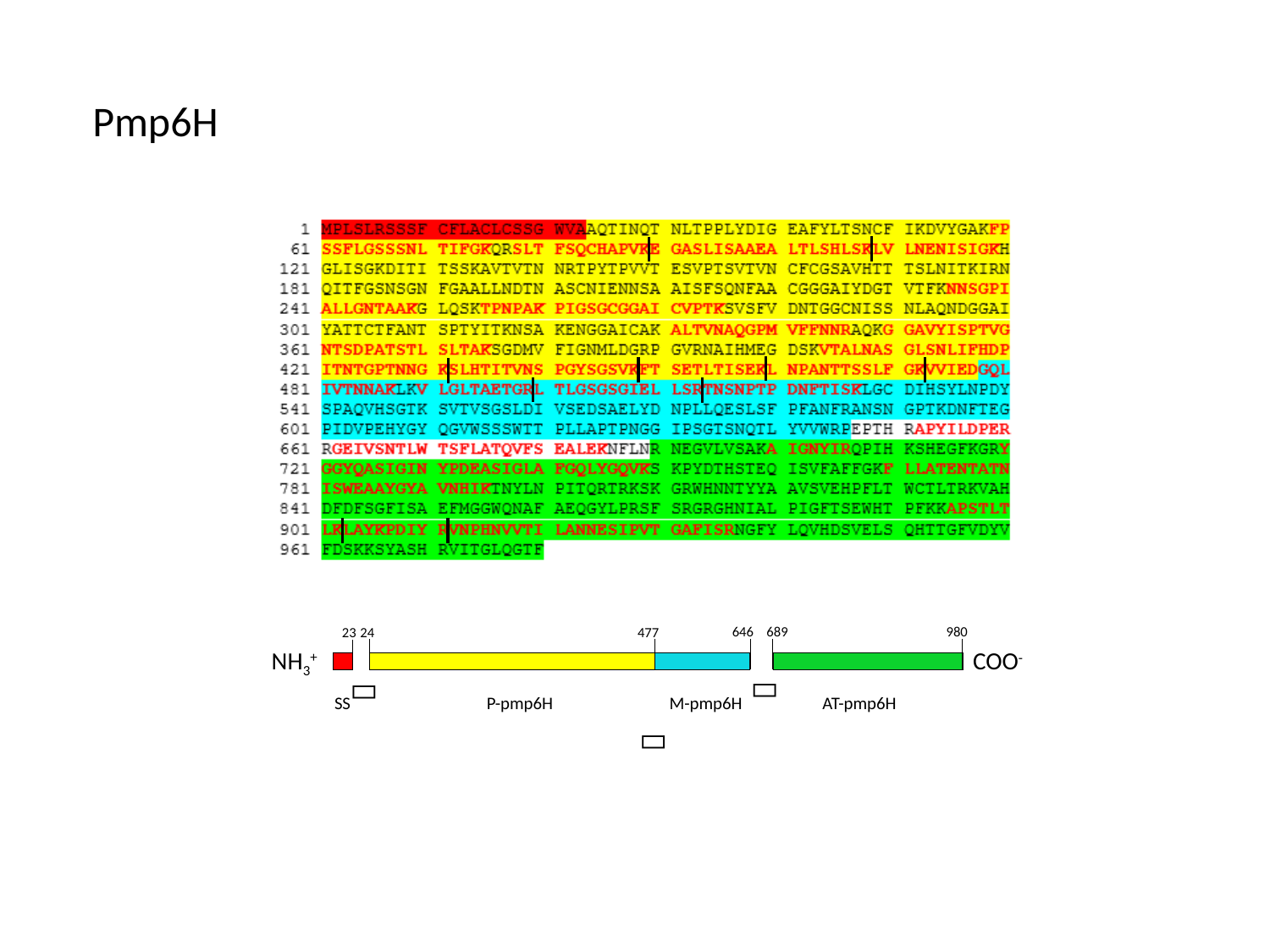

Pmp6H
980
689
646
477
23
24
NH3+
COO-


SS P-pmp6H M-pmp6H AT-pmp6H


## Slide 7
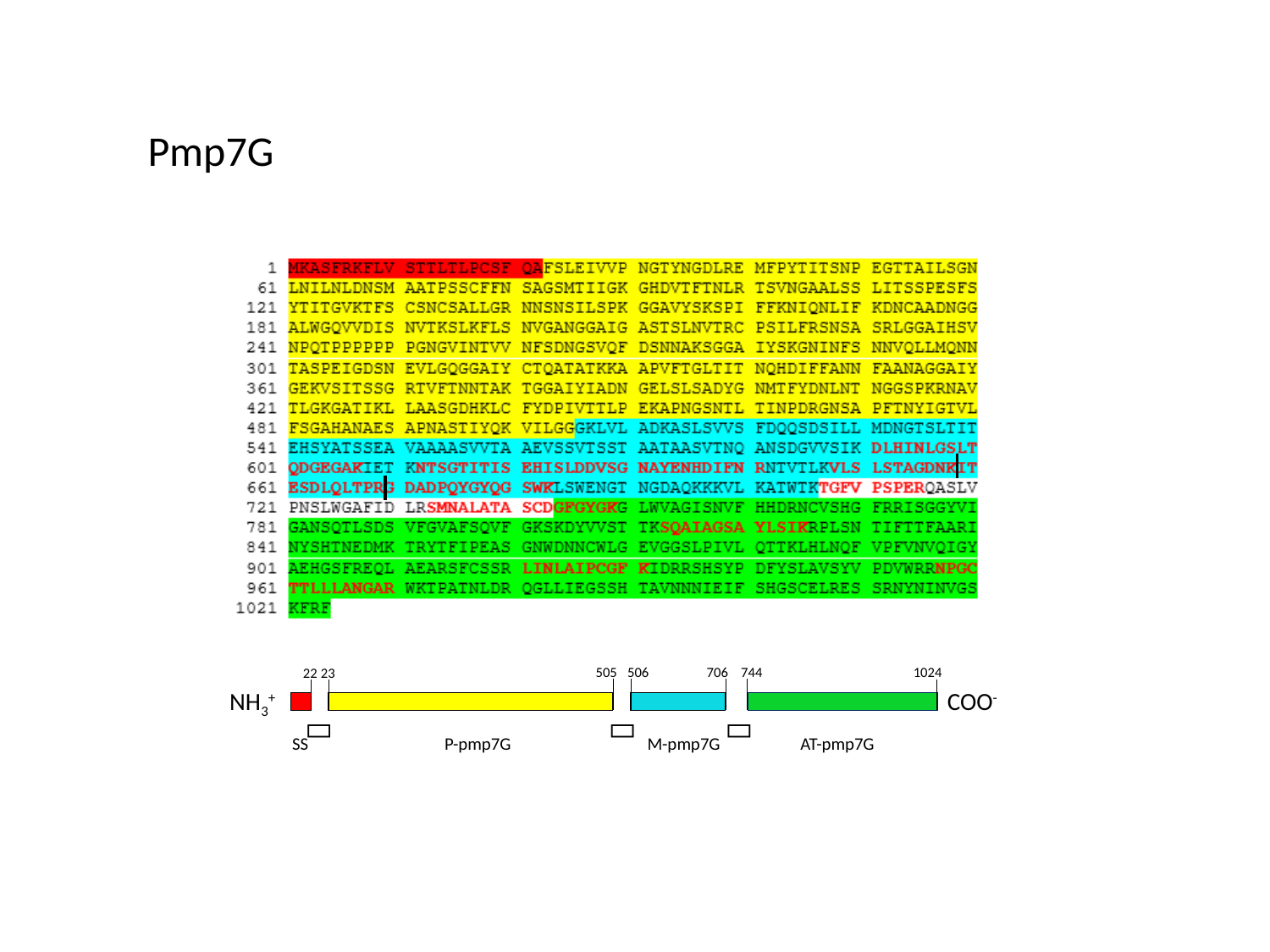

Pmp7G
1024
744
506
706
505
22
23
NH3+
COO-



SS P-pmp7G M-pmp7G AT-pmp7G

## Slide 8
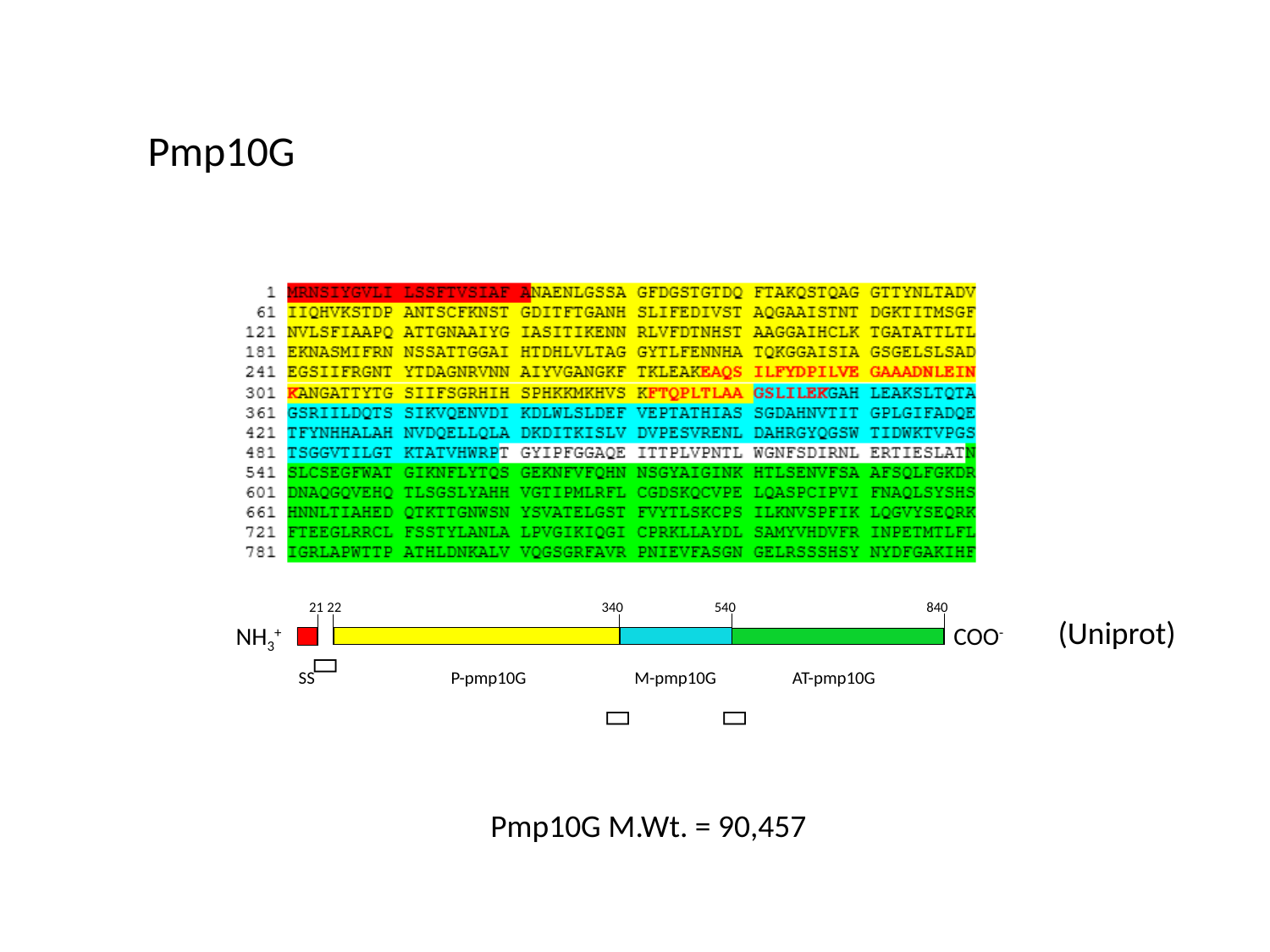

Pmp10G
840
540
340
21
22
NH3+
COO-

SS P-pmp10G M-pmp10G AT-pmp10G


(Uniprot)
Pmp10G M.Wt. = 90,457

## Slide 9
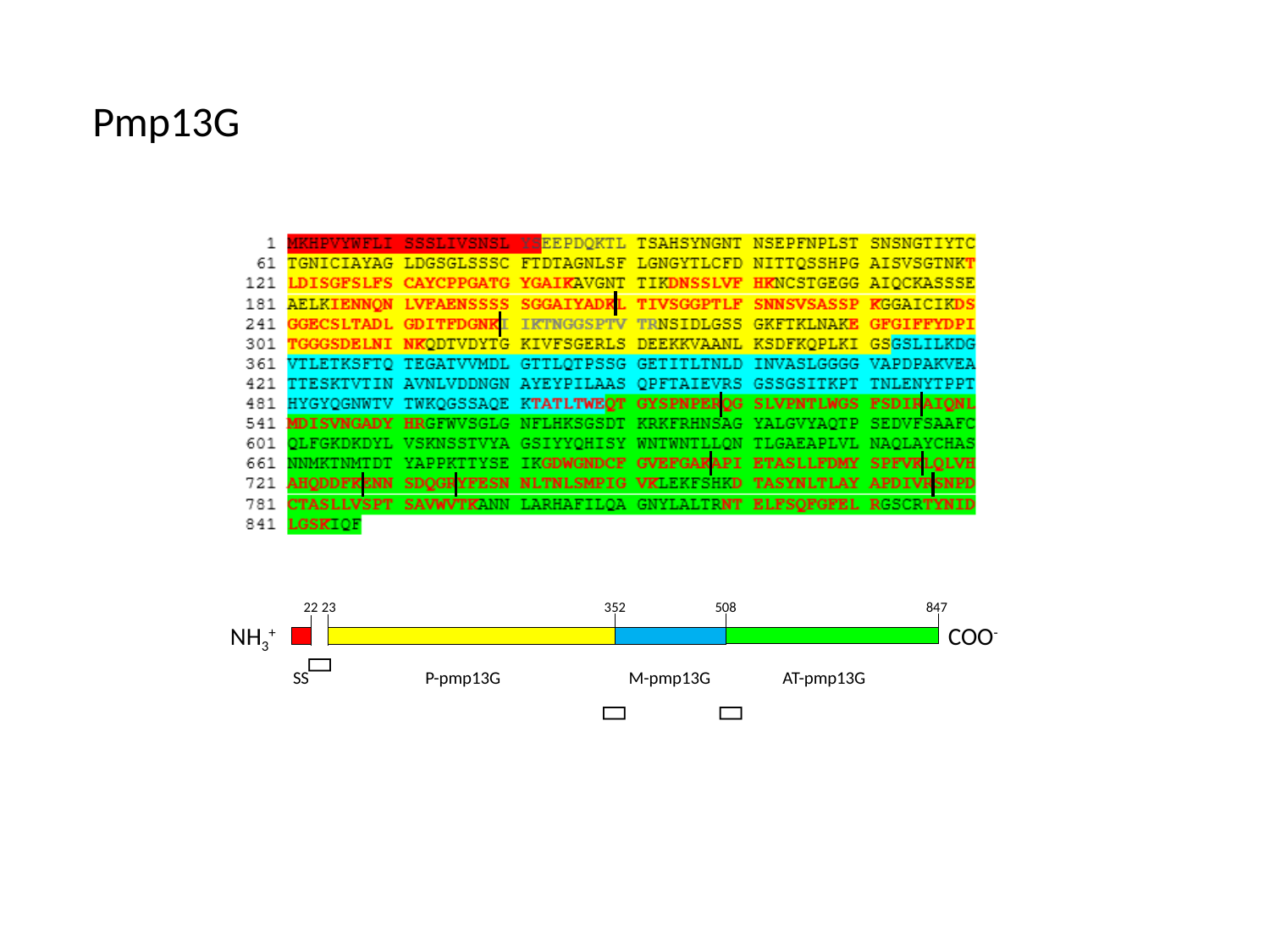

Pmp13G
847
508
352
22
23
NH3+
COO-

SS P-pmp13G M-pmp13G AT-pmp13G



## Slide 10
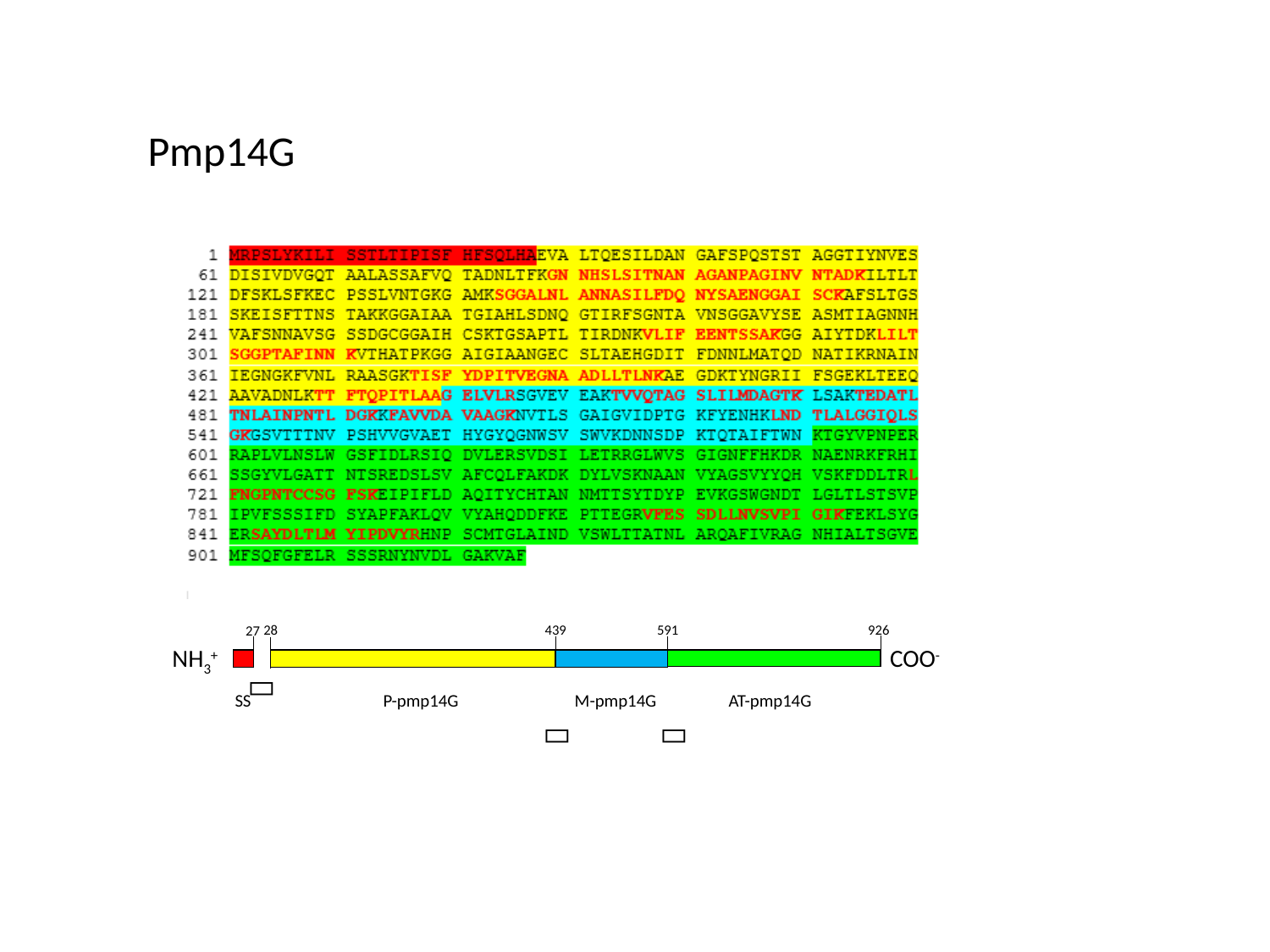

Pmp14G
926
591
439
28
27
NH3+
COO-

SS P-pmp14G M-pmp14G AT-pmp14G



## Slide 11
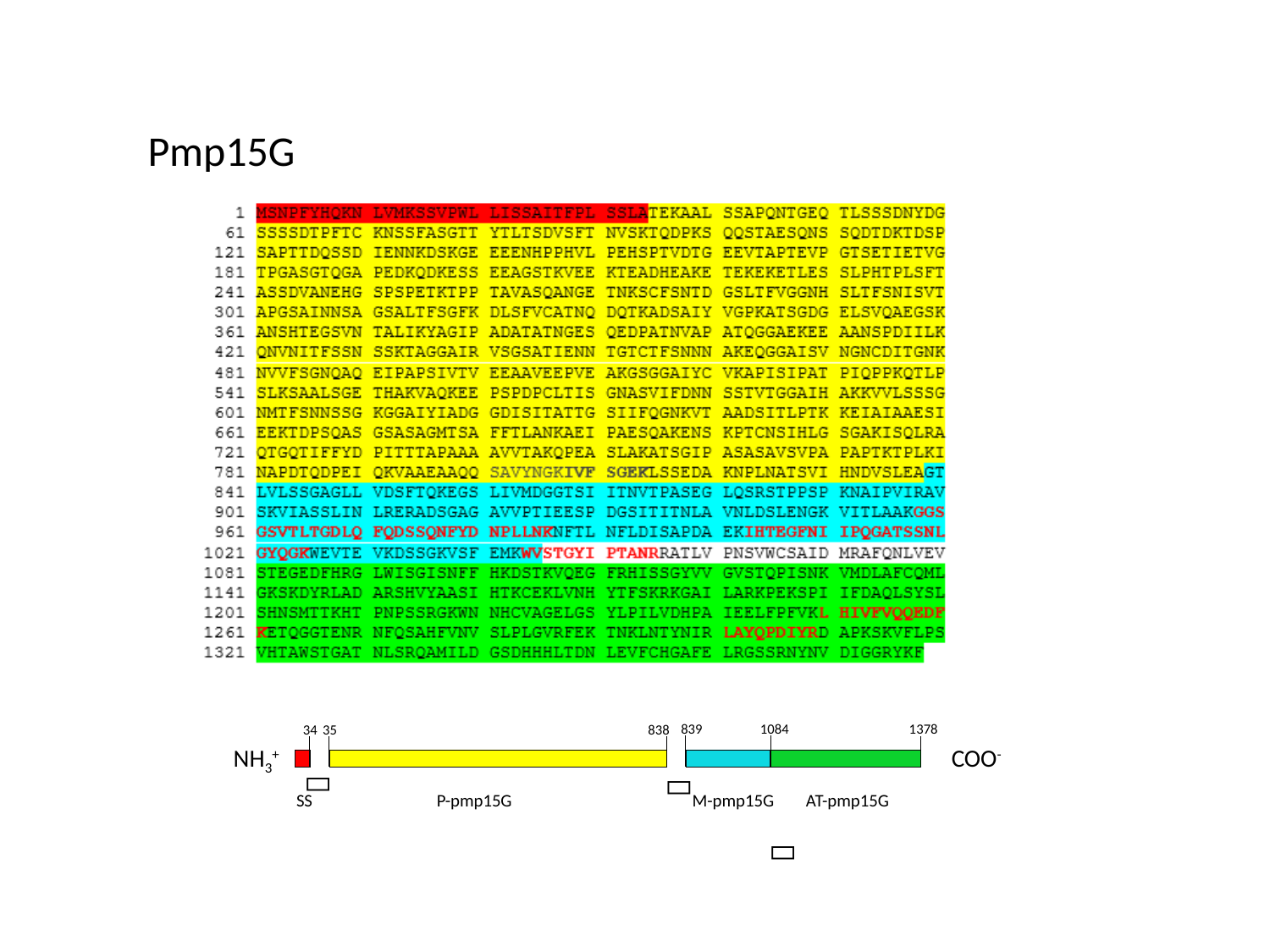

Pmp15G
1378
1084
839
838
34
35
NH3+
COO-


SS P-pmp15G M-pmp15G AT-pmp15G


## Slide 12
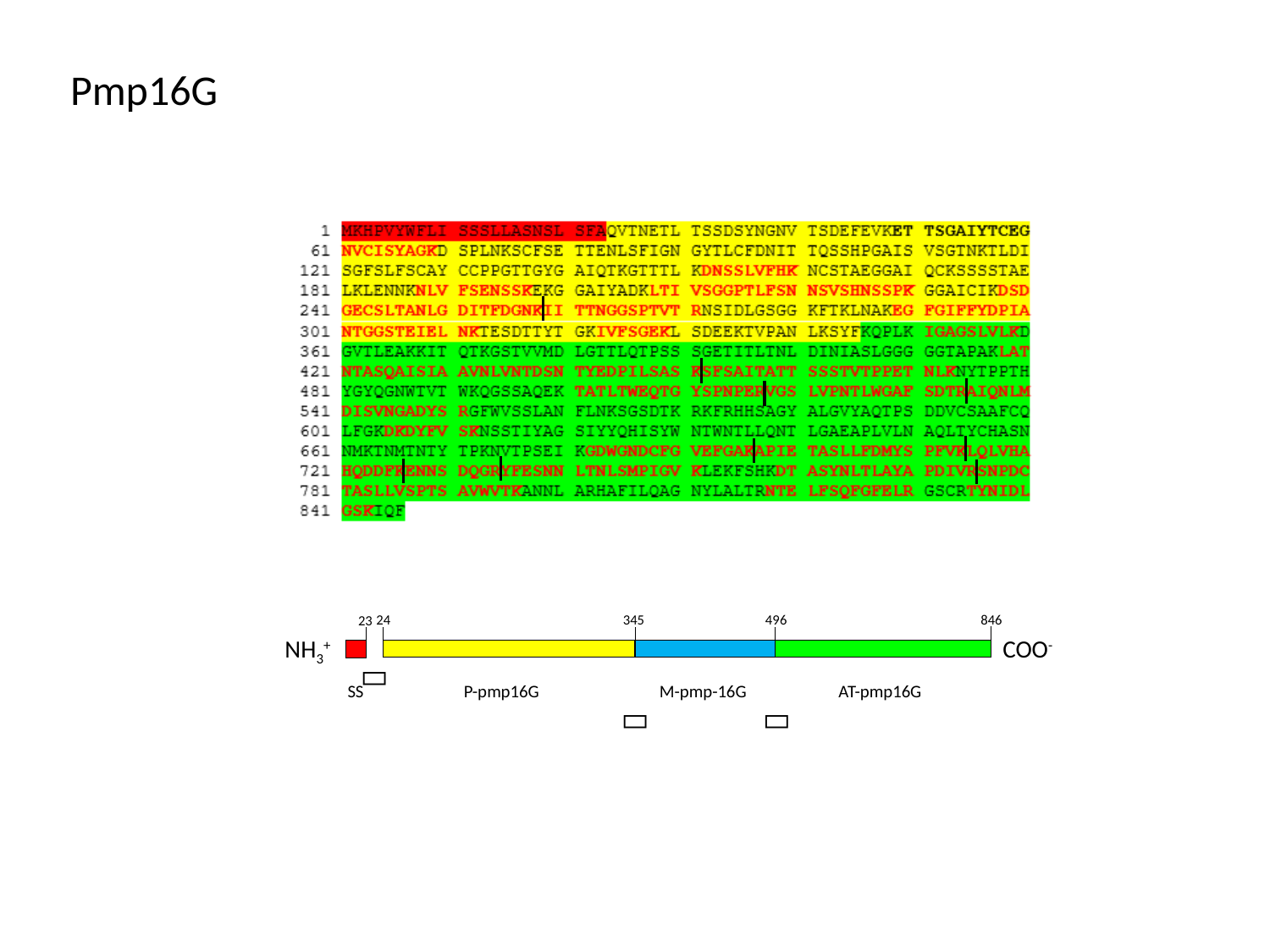

Pmp16G
846
496
345
24
23
NH3+
COO-

SS P-pmp16G M-pmp-16G AT-pmp16G



## Slide 13
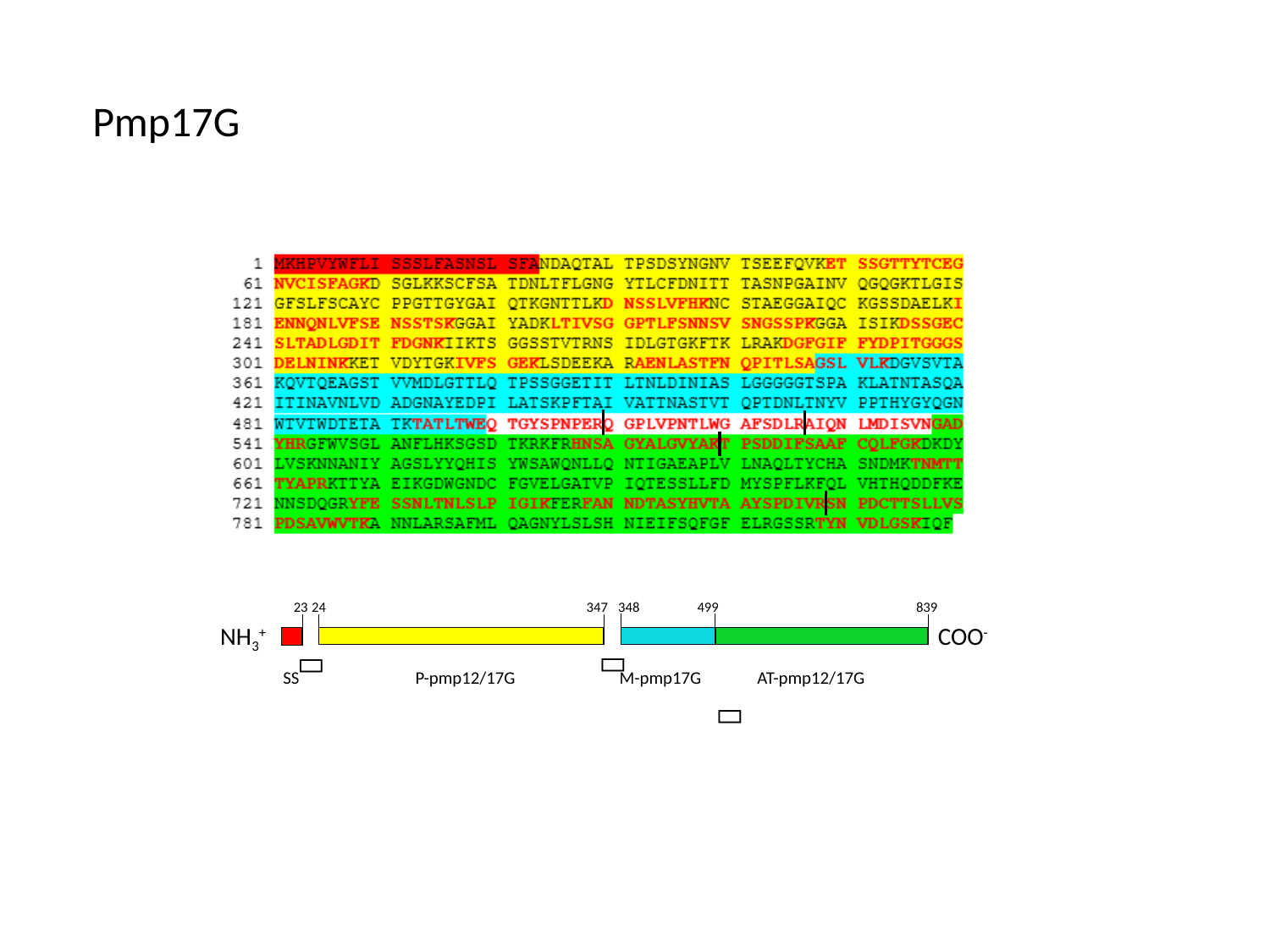

Pmp17G
839
348
499
347
23
24
NH3+
COO-


SS P-pmp12/17G M-pmp17G AT-pmp12/17G


## Slide 14
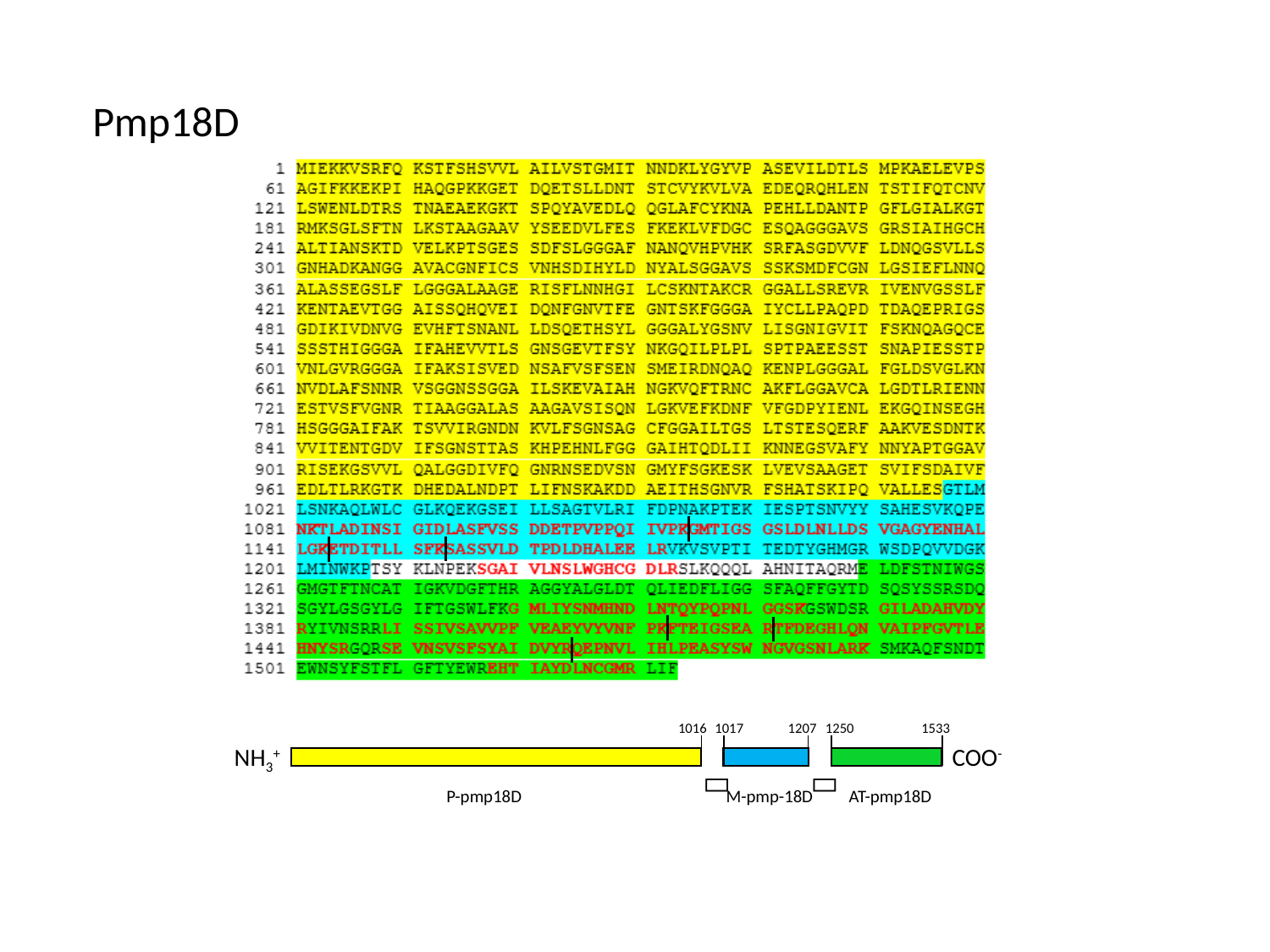

Pmp18D
1207
1533
1016
1017
1250
NH3+
COO-


P-pmp18D M-pmp-18D AT-pmp18D
