# Supplementary figures and images for: Proteomic characterisation of the Chlamydia abortus outer membrane complex (COMC) using combined rapid monolithic column liquid chromatography and fast MS/MS scanning
Source: PLoS One. 2019 Oct 24;14(10):e0224070. doi: 10.1371/journal.pone.0224070 (PMC6812762; doi:10.1371/journal.pone.0224070)

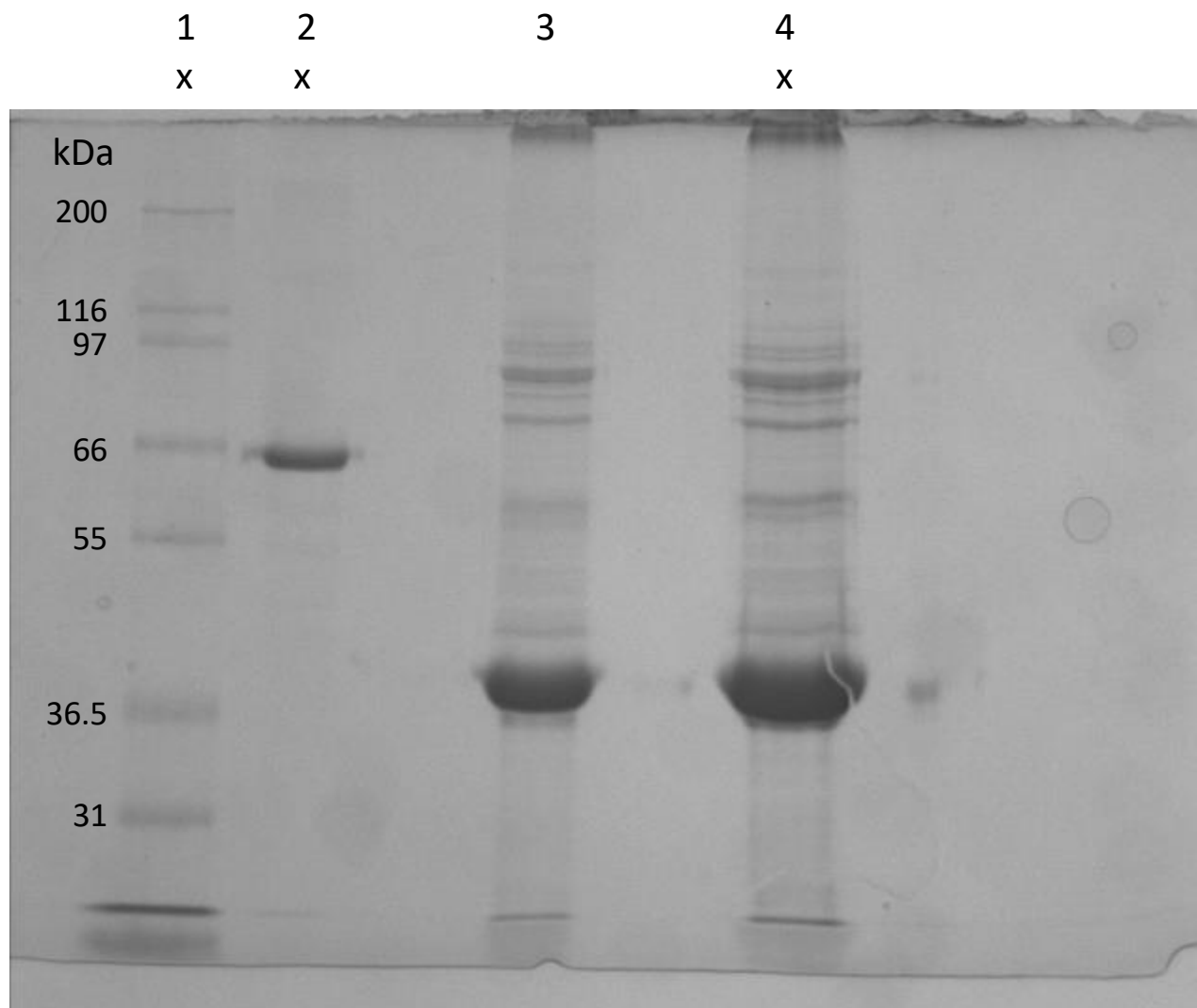

Supplement: S1 File — Original SDS-PAGE gel visualised with SimplyBlue Safestain and image captured on an AlphaImager 2200 (Alpha Innotech). Lanes: 1, Molecular Weight Markers (Mark12 Unstained Protein Standard, Invitrogen); 2, Bovine serum albumin (BSA); 3, COMC (10μg) sample; 4, COMC (20μg) sample. Lane 3 was used to create Fig 1. Lanes 1, 2 and 4 are not included as denoted by “X”. (PDF) [file pone.0224070.s002.pdf]
