# Supplementary material for: Proteomic characterisation of the Chlamydia abortus outer membrane complex (COMC) using combined rapid monolithic column liquid chromatography and fast MS/MS scanning
Source: PLoS One. 2019 Oct 24;14(10):e0224070. doi: 10.1371/journal.pone.0224070 (PMC6812762; doi:10.1371/journal.pone.0224070)
Supplement: S1 Table — (DOCX) [file pone.0224070.s003.docx]

**S1 Table. Proteins from *C. abortus* S26/3 COMC identified with one unique validated peptide.**

| Gene No. | Encoded protein | Predicted molecular mass (kDa) | Predicted cellular localization ^a^ | Protein coverage (%SC) ^b^ | Gel slice(s) ^c^ | Predicted protein processing ^d^ |
| --- | --- | --- | --- | --- | --- | --- |
| CAB167 | Hypo. Protein (TARP) | 91,404 | Unknown | 1 | 26 | C |
| CAB493 | DNA gyrase subunit B (gyrB) | 90,446 | Unknown | 1 | 26 | C |
| CAB884 | Put. phosphoenolpyruvate carboxykinase (pckG) | 66,780 | Unknown | 2.7 | 12 | UC |
| CAB304 | exodeoxyribonuclease VII large subunit (xseA) | 63,857 | Unknown | 1.6 | 13 | UC |
| CAB263 | Put. inner membrane protein | 63,377 | IM | 2.4 | 10 | UC |
| CAB806 | Put. cysteinyl-tRNA synthetase (cysS) | 54,563 | Cyto | 1.9 | 9 & 22/23 | C |
| CAB645 | Pyruvate kinase | 52,703 | Unknown | 3.5 | 14 | UC |
| CAB750 | Periplasmic serine endoprotease (DegP-like) | 52,122 | Periplasmic space | 2.5 | 14/15 | UC |
| CAB887 | Trigger factor (tig) | 49,716 | Cyto | 2.5 | 15 | UC |
| CAB835 | UDP-N-acetylmuramoyl-tripeptide--D-alanyl-D-alanine ligase | 49,247 | Cyto | 2.5 | 14 | UC |
| CAB036 | Put. type III secretion or flagellar-type ATP synthase | 48,283 | Cyto | 2.5 | 15 | UC |
| CAB932 | Enolase (eno) | 45,463 | Unknown | 3.5 | 14 | UC |
| CAB840 | Put. nucleosidase | 33,433 | Unknown | 5.5 | 21 | UC |
| CAB637 | Hypo. protein | 29,273 | Unknown | 6.9 | 22 | UC |
| CAB052 | ABC transporter ATP-binding protein | 28,688 | Cyto | 9.8 | 23 | UC |
| CAB135 | Put. transport lipoprotein | 28,007 | Unknown | 4.8 | 23 | UC |
| CAB045 | Uridylate kinase (pyrH) | 26,609 | Cyto | 10.6 | 22 | UC |
| CAB094 | 50S ribosomal protein L4 (rplD) | 25,006 | Unknown | 3.1 | 23 | UC |
| CAB546 | hypothetical protein | 23,693 | Unknown | 9.7 | 26 | UC |
| CAB337 | Superoxide dismutase | 23,648 | Unknown | 10.6 | 25 | UC |
| CAB198 | Thiamine-phosphate synthase (thiE) | 22,773 | Unknown | 12.3 | 24 | UC |
| CAB396 | Single-stranded DNA-binding protein | 17,573 | Unknown | 10.1 | 25 | UC |
| CAB105 | **30S ribosomal protein S8 (rpsH)** | 15,219 | Unknown | 6.8 | 25 | UC |
| CAB112 | 30S ribosomal protein S11 (rpsK) | 13,884 | Unknown | 11.4 | 25 | UC |
| CAB627A | **30S ribosomal protein S16 (rpsP)** | 13,876 | Unknown | 8.4 | 25 | UC |
| CAB102 | 50S ribosomal protein L14 (**rplN)** | 13,459 | Unknown | 10.7 | 25 | UC |

^a^ Cyto, cytoplasmic; OM, outer membrane; Extra, extracellular; Unknown, indicates no predicted location.

^b^ Protein coverage is expressed as a percentage of total sequence coverage (%SC).

^c^ The gel slice that the peptides were detected in (*cf* Fig 1).

^d^ Indicates whether location of peptides in a particular slice is in agreement with the expected location of the uncleaved (UC) mature protein or is suggestive of potential post-translational cleavage (C).
